# Supplementary material for: Chromatin remodeling by Pol II primes efficient Pol III transcription
Source: Nat Commun. 2023 Jun 16;14:3587. doi: 10.1038/s41467-023-39387-4 (PMC10276017; doi:10.1038/s41467-023-39387-4)
Supplement: Supplementary file 1 — Supplementary Information [file 41467_2023_39387_MOESM1_ESM.pdf]

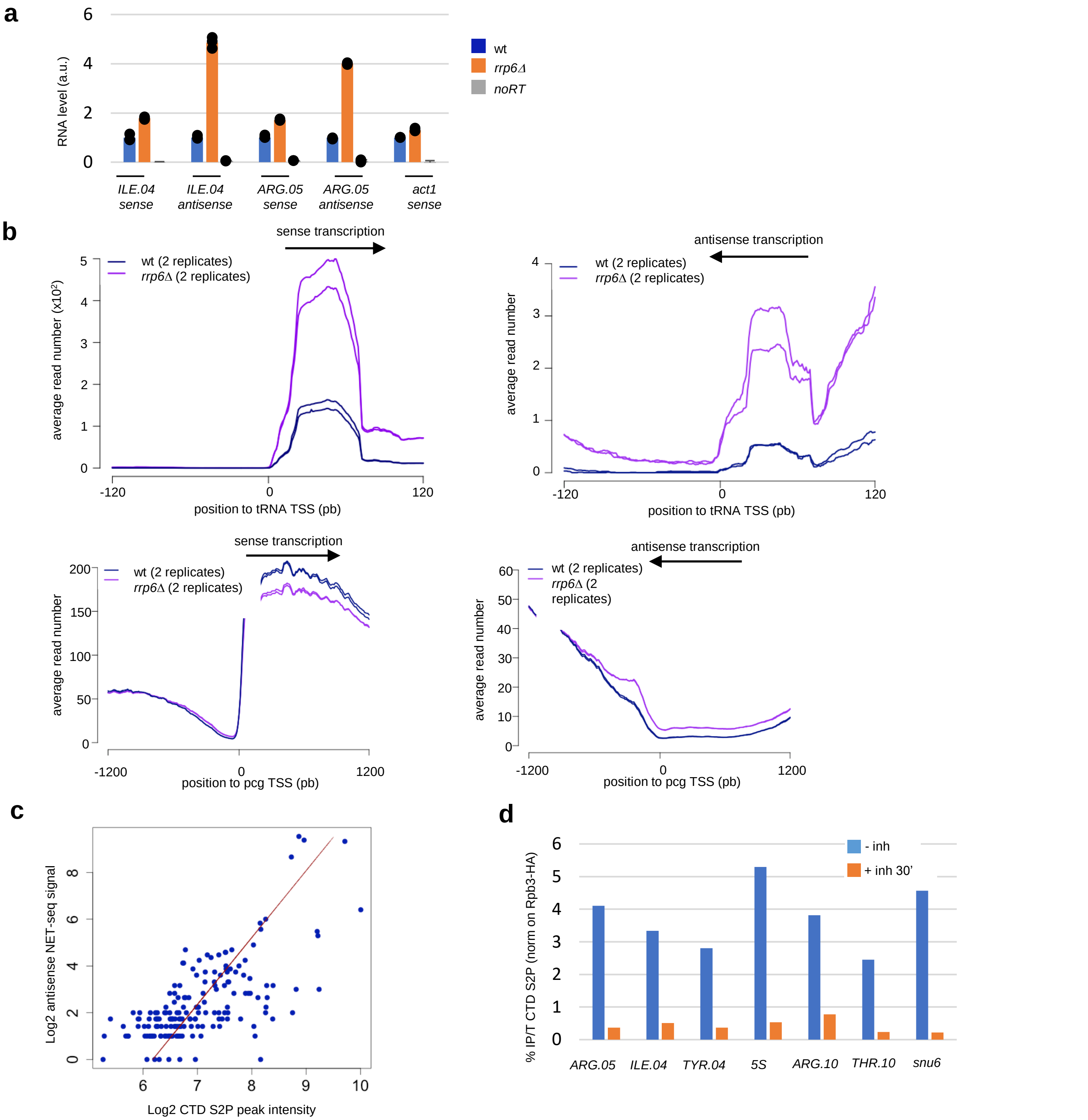

Supplementary Figure 1. A. Strand-specific RT-qPCR analysis of the indicated loci. Relative level of each transcript in the wt was set to 1. Mean and SEM values were calculated from three biological replicates. *noRT* indicates that the reaction was performed in the absence of reverse transcriptase. **B**. Metagene showing the 5% trimmed mean read coverage in sense (left panel) or antisense (right panel) around tRNA genes (top panel) or protein coding genes (pcg, bottom panel) aligned to their transcription start site (0). Two biological replicates of wild-type (blue) and Δ*rrp6* (purple) are shown. Total RNA-seq data from GSE104713.**C**. Log-log relationship between the level of Pol II-specific antisense transcription (NET-seq) and of CTD S2P at tDNAs. Linear regression is very significant (*p-value* < 2.2 x 10-16) with a R^2 coefficient of 0.45. **D**. ChIP experiment performed on chromatin prepared from the Rpb3-HA Lsk1-as strain grown in the absence (- inh) or in the presence (+ inh 30') of 10 µmol 3-MB-PP1 inhibitor and an untagged control using anti-HA and anti CTD-S2P antibodies. The amplicons targeted the indicated class III loci. Each column represents the CTD S2P value normalized on the Rpb3-HA value (n = 3 biological replicates). Source data, including the unnormalized data used to make the figure, are provided as a Source Data file.

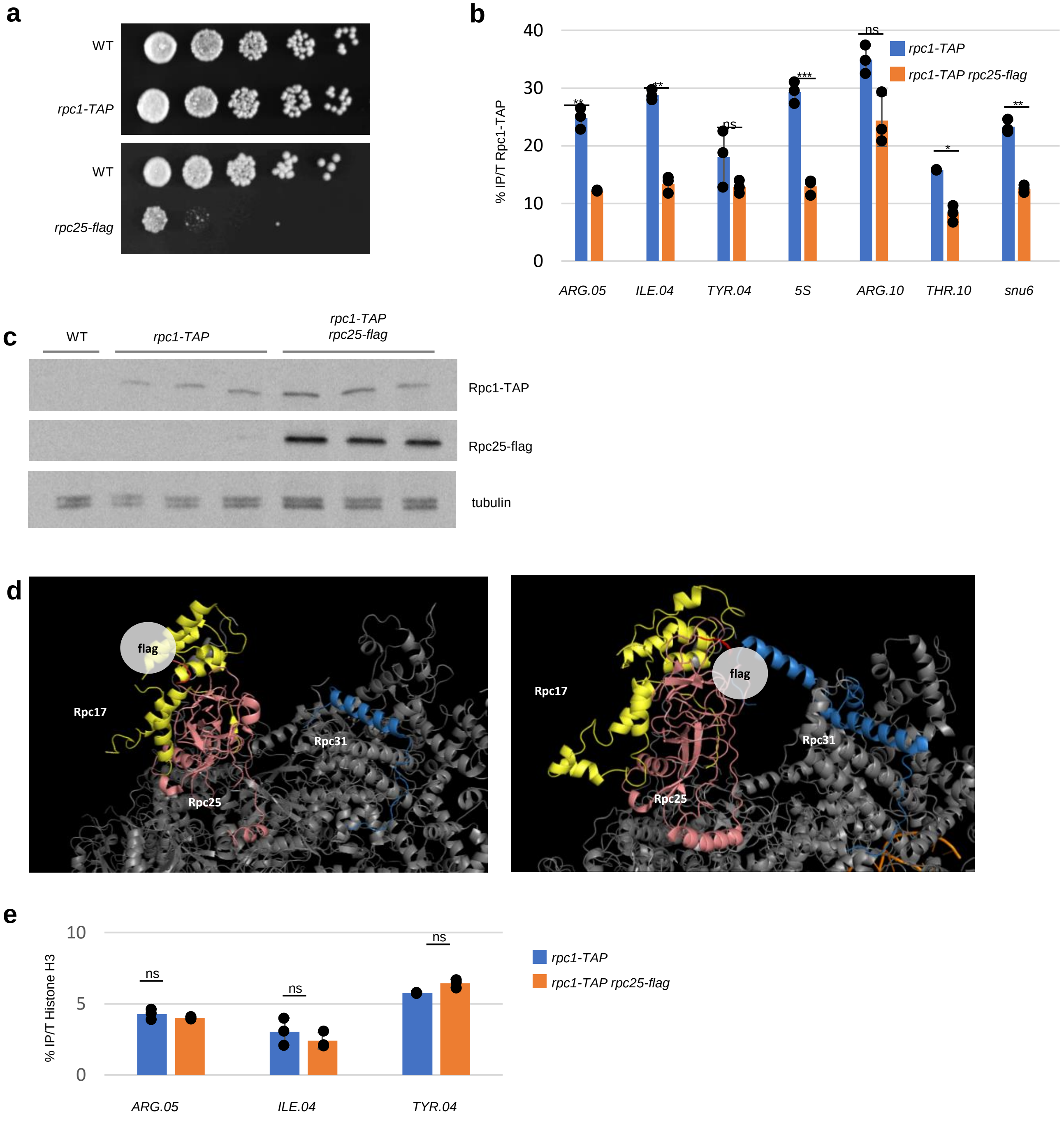

Supplementary Figure 2.
A. Growth assay of the indicated strains. Exponential growth cultures were diluted (10-fold dilutions per spot), spotted on YES medium and incubated for 3 days at 32°C. **B**. ChIP experiment performed on chromatin prepared from the indicated strains using anti-TAP antibody. The amplicons targeted the indicated class III loci. Each column represents the averaged value and error bars are the standard deviation (n = 3 biological replicates), * $p < 0.05$, ** $p < 0.01$, *** $p < 0.001$; ns not significant upon paired t-test. Source data are provided as a Source Data file. **C.** Western blot analysis of the level of Rpc1-TAP and Rpc25-flag in the indicated strains. Whole cell protein extracts (using the TCA protocol) from biological triplicates were separated by PAGE and probed using anti-TAP, anti-flag and anti-tubulin as loading control. Source data including uncropped images and size ladder are provided as a Source Data file. **D**. Left panel. A region of the apo Pol III open complex structure (protein databank accession number: pdb 6EU2) (Abascal-Palacios et al., 2018) showing Rpc17 (yellow), Rpc25 (pink) and Rpc31 (blue). The 5x flag fusion peptide of the Rpc25-flag subunit is indicated at the C-terminal end of Rpc25. Right panel. Same as in the left panel except that the Pol III pre-initiation complex structure is shown with the bound DNA (orange). **E**. ChIP experiment performed on chromatin prepared from the indicated strains using anti-H3 antibody. The amplicons targeted the indicated class III loci. Each column represents the averaged value and error bars are the standard deviation (n = 3 biological replicates), * $p < 0.05$, ** $p < 0.01$, *** $p < 0.001$; ns not significant upon paired t-test. Source data are provided as a Source Data file.

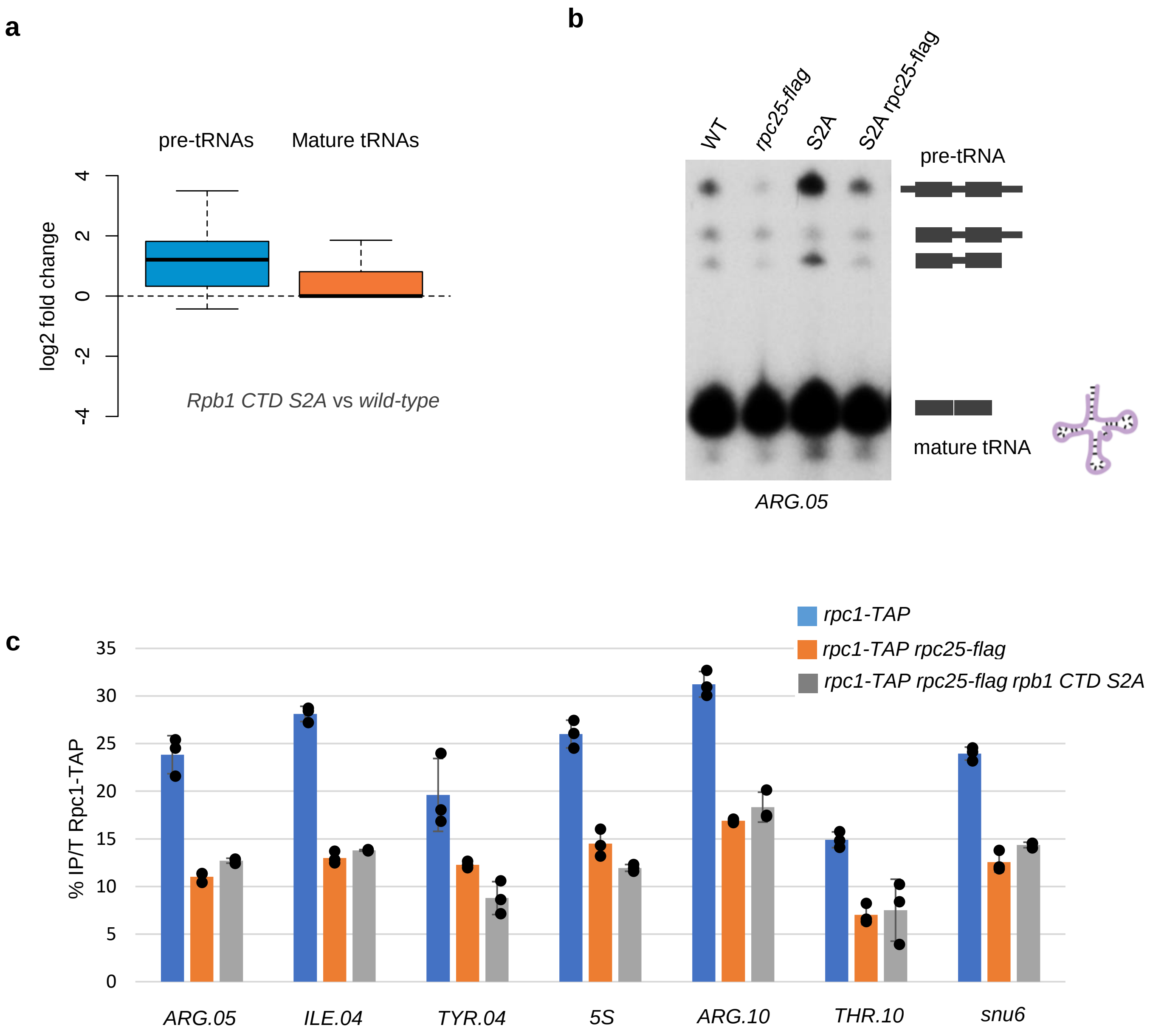

Supplementary Figure 3. A. Boxplot of the log2 fold-change for pre- or mature tRNAs (n = 171 tDNA loci in Fission yeast) from RNA- seq in the indicated strains. **B.** tRNA northern blot probing SPBTRNAARG.05 (*ARG.05*) from the indicated strains. A cartoon representation of the various tRNA processed forms is shown on the right from top to bottom: full pre-tRNA, 5'-spliced pre-tRNA, 3'- spliced pre-tRNA, mature tRNA. Source data including uncropped images are provided as a Source Data file. **C**. ChIP experiment performed on chromatin prepared from the indicated strains using anti-TAP antibody. The amplicons targeted the indicated class III loci. Each column represents the averaged value and error bars are the standard deviation (n = 3 biological replicates), * $p < 0.05$, ** $p < 0.01$, *** $p < 0.001$; ns not significant upon paired t-test. Source data are provided as a Source Data file.

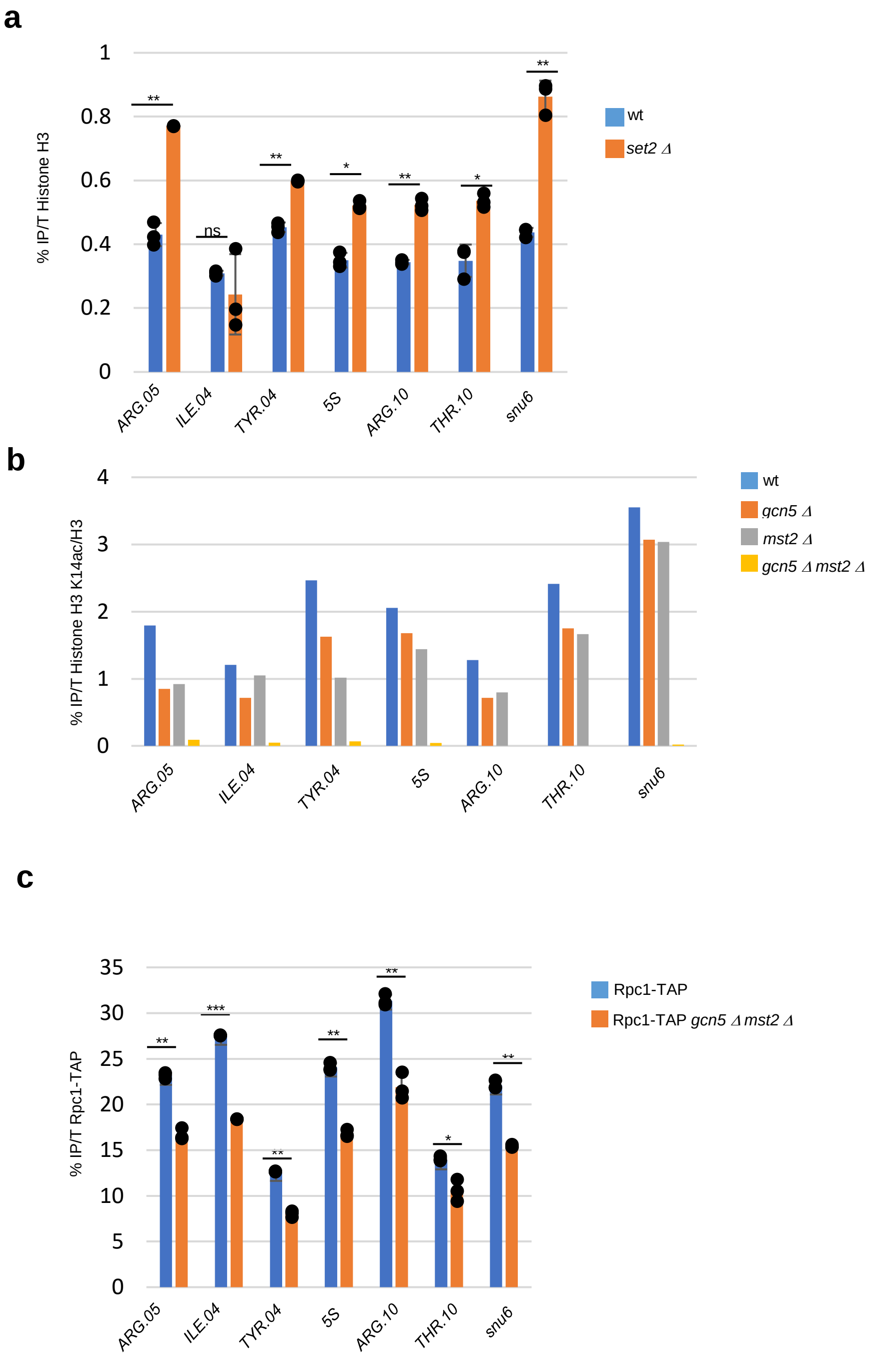

Supplementary Figure 4. A. ChIP experiment performed on chromatin prepared from the indicated strains using anti-H3 antibody. The amplicons targeted the indicated class III loci. Each column represents the averaged value and error bars are the standard deviation (n = 3 biological replicates), * $p < 0.05$, ** $p < 0.01$, *** $p < 0.001$; ns not significant upon paired t-test. Source data are provided as a Source Data file. **B**. ChIP experiment performed on chromatin prepared from the indicated strains using anti-H3 and anti CTD-H3K14ac antibodies. The amplicons targeted the indicated class III loci. Each column represents the H3K14ac value normalized on the H3 value (n = 3 biological replicates). Source data, including the unnormalized data used to make the figure, are provided as a Source Data file.**C**. ChIP experiment performed on chromatin prepared from the indicated strains using anti-TAP antibody. The amplicons targeted the indicated class III loci. Each column represents the averaged value and error bars are the standard deviation (n = 3 biological replicates), * $p < 0.05$, ** $p < 0.01$, *** $p < 0.001$; ns not significant upon paired t-test. Source data are provided as a Source Data file.

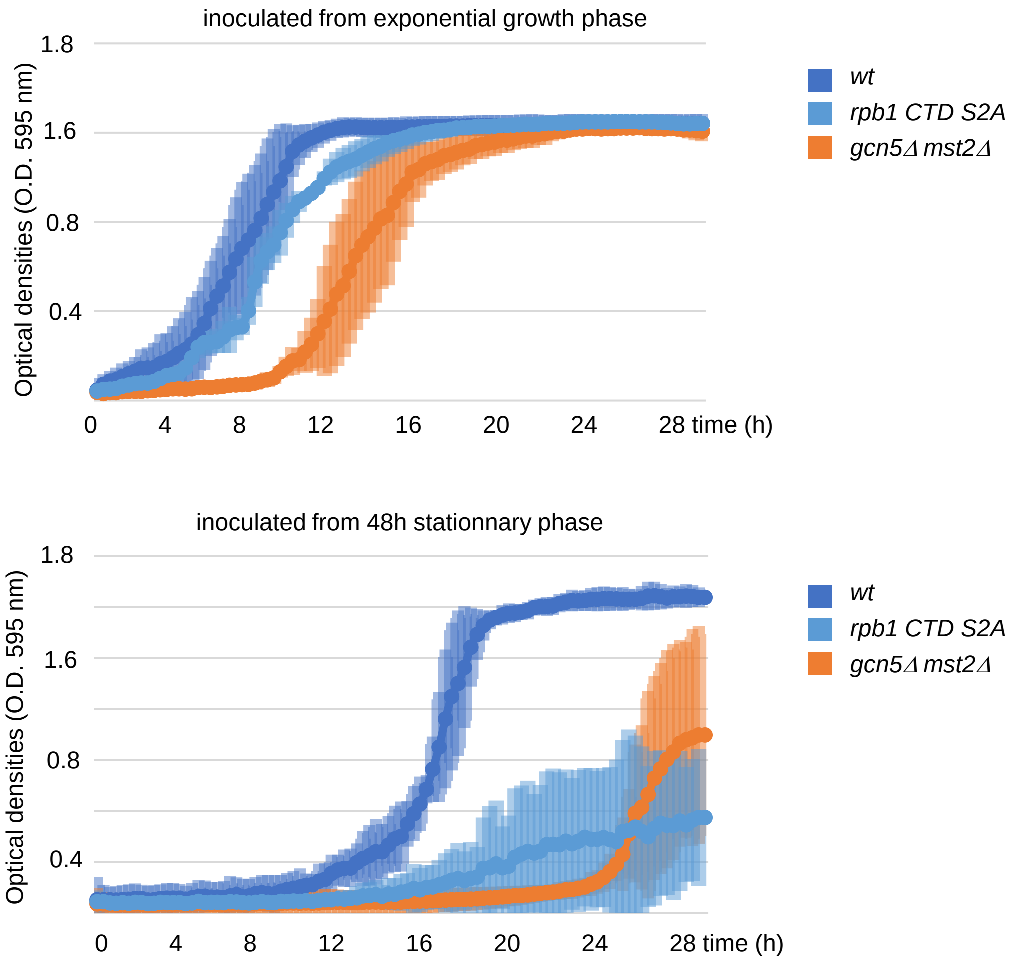

Supplementary Figure 5. Growth assay at 32°C of the indicated strains spotted from exponential growth phase cultures (Top panel) or from cultures maintained in stationary phase (bottom panel) for 48 hours. Optical densities at 595 nm were measured every 10 minutes for 28 hours. Data are presented as the averaged value ± SEM (n = 3 biological replicates).
