# Supplementary material for: Chromatin remodeling by Pol II primes efficient Pol III transcription
Source: Nat Commun. 2023 Jun 16;14:3587. doi: 10.1038/s41467-023-39387-4 (PMC10276017; doi:10.1038/s41467-023-39387-4)
Supplement: Supplementary file 3 — Description of Additional Supplementary Files [file 41467_2023_39387_MOESM3_ESM.pdf]

Description of Additional Supplementary Files

File Name: Supplementary Data 1
Description: Strains and primers used in this study.
