# Supplementary material for: Chromatin remodeling by Pol II primes efficient Pol III transcription
Source: Nat Commun. 2023 Jun 16;14:3587. doi: 10.1038/s41467-023-39387-4 (PMC10276017; doi:10.1038/s41467-023-39387-4)
Supplement: Supplementary file 6 — Source Data [file 41467_2023_39387_MOESM6_ESM.zip › Source data file/figureS3c/2017_06_19_arg05_lsk1_rrp6_S2A_with_rpc25.pptx]

## Slide 1
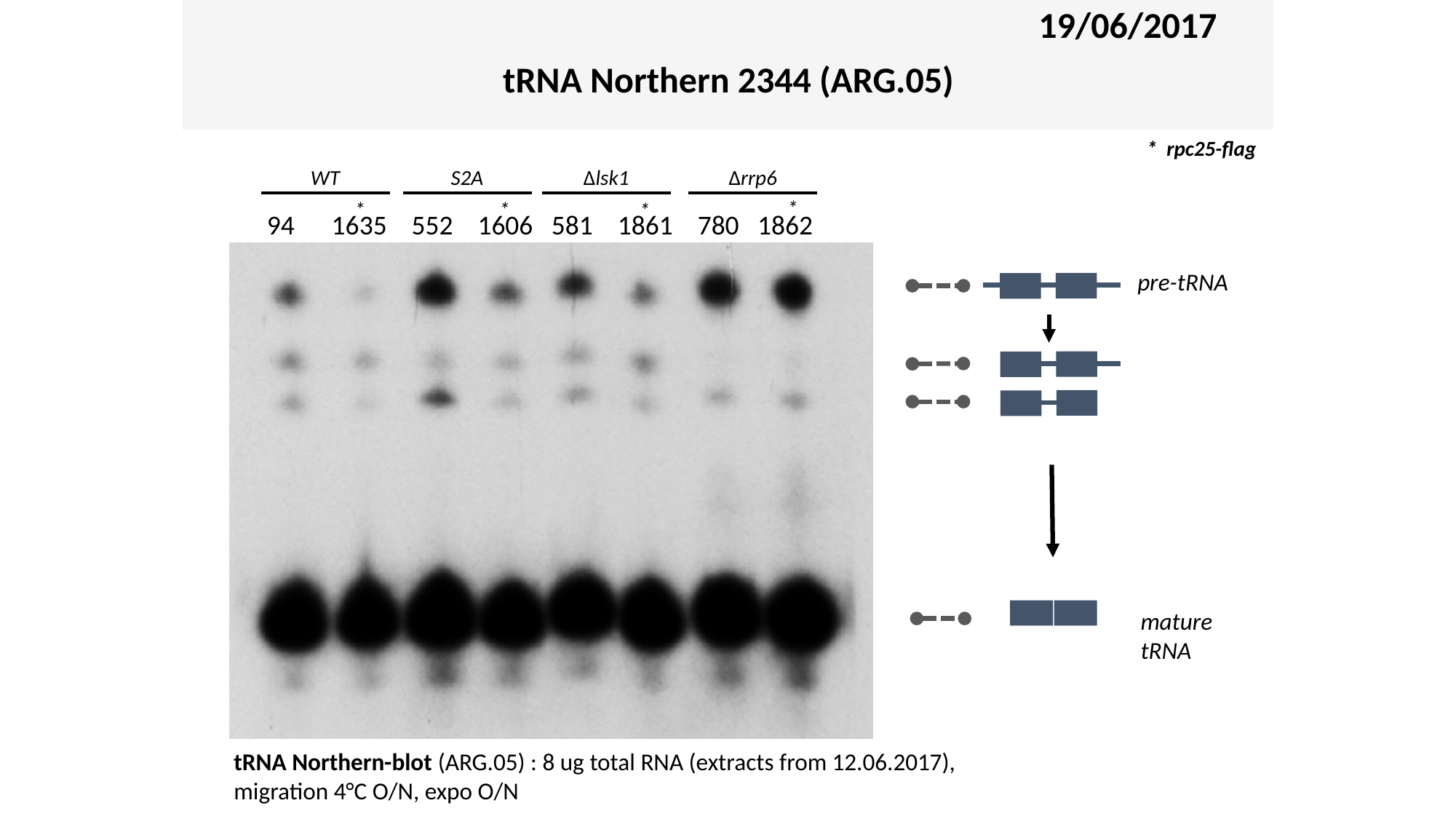

19/06/2017
tRNA Northern 2344 (ARG.05)
* rpc25-flag
∆lsk1
WT
S2A
∆rrp6
*
*
*
*
94 1635 552 1606 581 1861 780 1862
pre-tRNA
mature tRNA
tRNA Northern-blot (ARG.05) : 8 ug total RNA (extracts from 12.06.2017), migration 4°C O/N, expo O/N
