# Supplementary figures and images for: Chromatin remodeling by Pol II primes efficient Pol III transcription
Source: Nat Commun. 2023 Jun 16;14:3587. doi: 10.1038/s41467-023-39387-4 (PMC10276017; doi:10.1038/s41467-023-39387-4)

## Slide 1
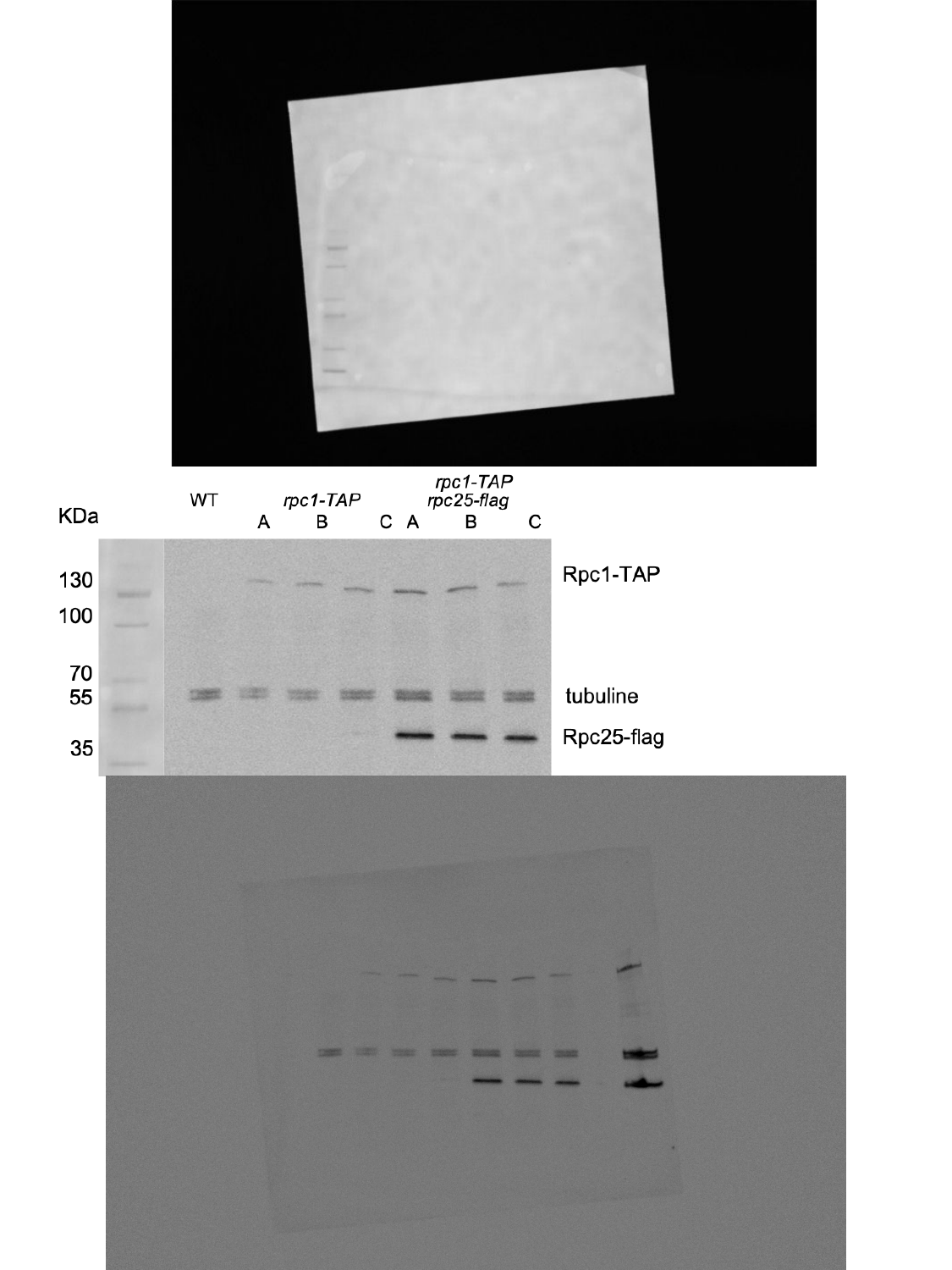

Supplement: Supplementary file 6 — Source Data [file 41467_2023_39387_MOESM6_ESM.zip › Source data file/Source Data Sup Fig 2C.pptx]

## Slide 1
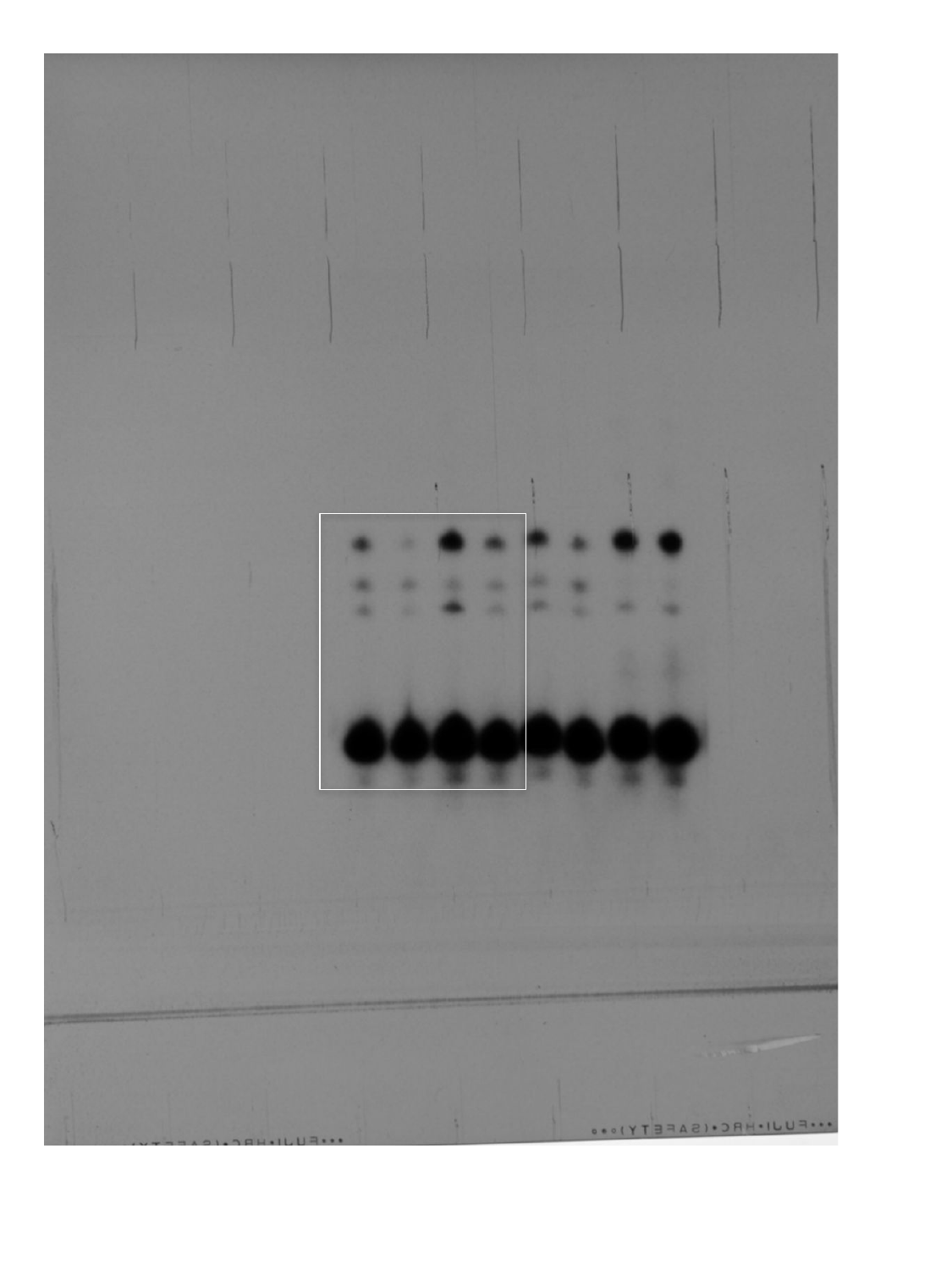

Supplement: Supplementary file 6 — Source Data [file 41467_2023_39387_MOESM6_ESM.zip › Source data file/Source Data Sup Fig 3C.pptx]

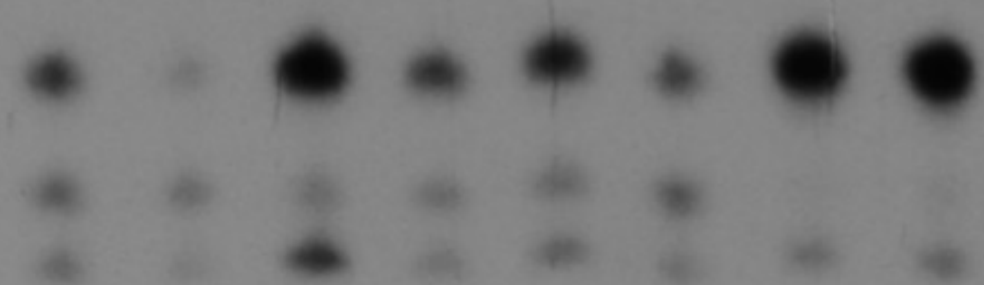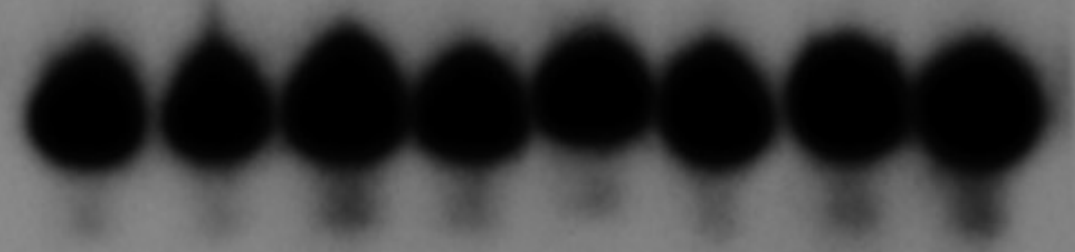

Supplement: Supplementary file 6 — Source Data [file 41467_2023_39387_MOESM6_ESM.zip › Source data file/figureS3c/2017_06_19_arg05_lsk1_rrp6_S2A_with_rpc25.pdf]

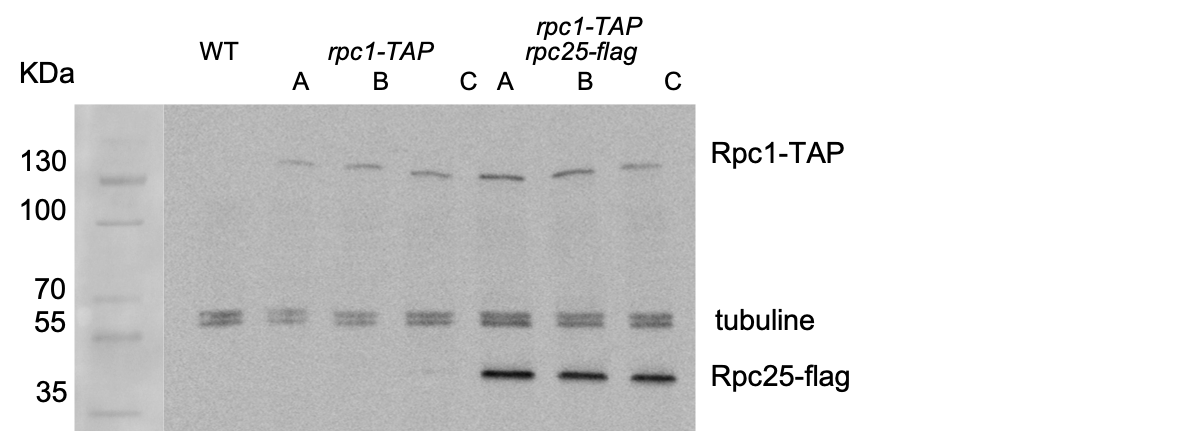

Supplement: Supplementary file 6 — Source Data [file 41467_2023_39387_MOESM6_ESM.zip › Source data file/figureS2c/wb_ysaline_monunted.png]

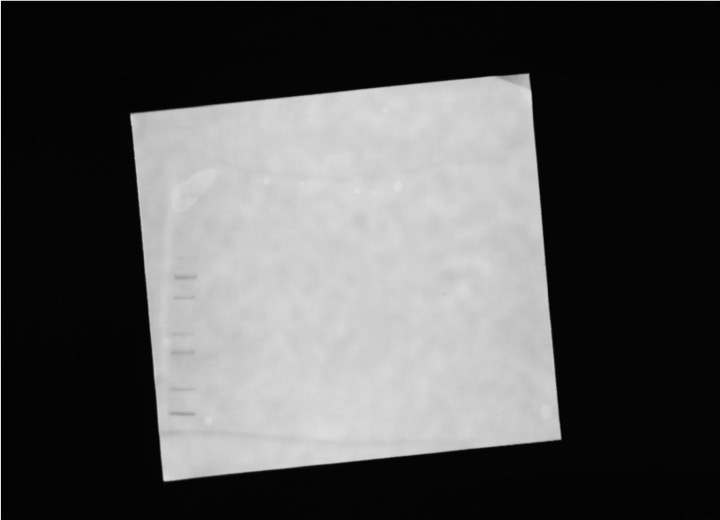

Supplement: Supplementary file 6 — Source Data [file 41467_2023_39387_MOESM6_ESM.zip › Source data file/figureS2c/wb_ysaline_ladder.tiff]

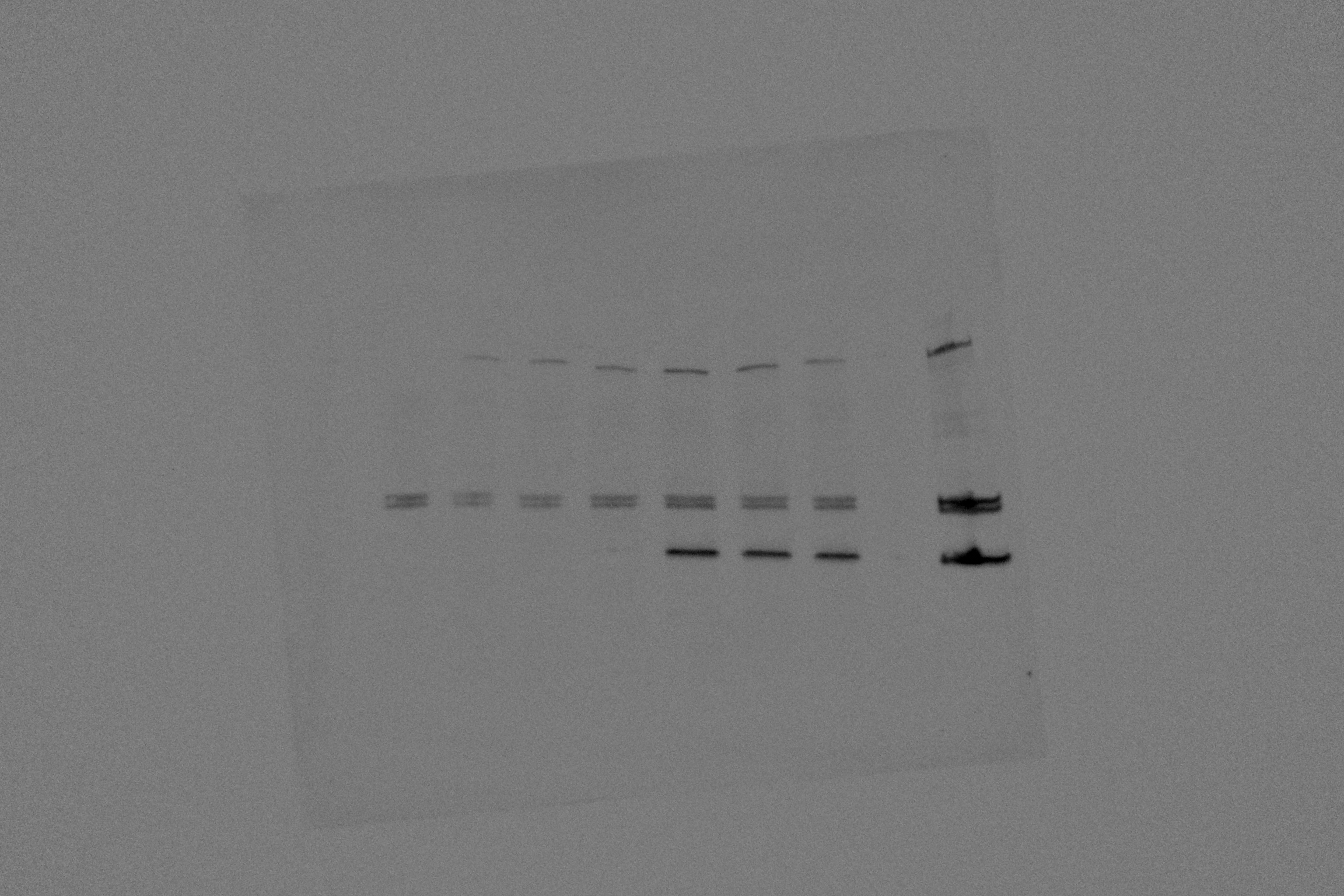

Supplement: Supplementary file 6 — Source Data [file 41467_2023_39387_MOESM6_ESM.zip › Source data file/figureS2c/wb_ysaline_rcp1-tap_rpc25_flag.tiff]
